# A virus-induced gene silencing method to study soybean cyst nematode parasitism in *Glycine max*

**DOI:** 10.1186/1756-0500-6-255

**Published:** 2013-07-06

**Authors:** Pramod K Kandoth, Robert Heinz, Greg Yeckel, Nathan W Gross, Parijat S Juvale, John Hill, Steven A Whitham, Thomas J Baum, Melissa G Mitchum

**Affiliations:** 1 Division of Plant Sciences, Christopher S. Bond Life Sciences Center and Interdisciplinary Plant Group, University of Missouri, Columbia, MO 65211, USA; 2 Department of Plant Pathology and Microbiology, Iowa State University, Ames, IA 50011, USA; 3 Current address: Pioneer Hi-Bred, Johnston IA, 50131, USA; 4 Current address: Beadle Center for Biotechnology, University of Nebraska, Lincoln NE, 68588, USA

**Keywords:** *Heterodera glycines*, Resistance, SCN, Soybean, Cyst nematode, Resistance, Virus-induced gene silencing, VIGS

## Abstract

**Background:**

*Bean pod mottle virus* (BPMV) based virus-induced gene silencing (VIGS) vectors have been developed and used in soybean for the functional analysis of genes involved in disease resistance to foliar pathogens. However, BPMV-VIGS protocols for studying genes involved in disease resistance or symbiotic associations with root microbes have not been developed.

**Findings:**

Here we describe a BPMV-VIGS protocol suitable for reverse genetic studies in soybean roots. We use this method for analyzing soybean genes involved in resistance to soybean cyst nematode (SCN). A detailed SCN screening pipeline is described.

**Conclusions:**

The VIGS method described here provides a new tool to identify genes involved in soybean-nematode interactions. This method could be adapted to study genes associated with any root pathogenic or symbiotic associations.

## Background

Virus-induced gene silencing (VIGS) is a reverse genetic tool that exploits RNA-mediated antiviral defense strategies used by plants [1]. VIGS is based on the formation of double-stranded RNA intermediates and structured single-stranded RNAs, which are formed during virus infection [2]. DICER-like ribonucleases digest the dsRNAs to produce 21-22 nucleotide long small interfering RNAs (siRNAs). These siRNAs, as part of RNA Induced Silencing Complex (RISC), lead to sequence-specific degradation of viral RNAs [3]. When a portion of an endogenous gene is inserted in the viral genome and introduced into plants, siRNAs specific to the endogenous gene are produced and lead to targeted degradation of endogenous gene transcripts [1,4]. VIGS has several advantages over other reverse genetic tools. For one, VIGS produces rapid and transient phenotypes. In addition, the methodology is relatively inexpensive and does not require the development of plant transformation protocols. VIGS is also particularly useful for functional studies of genes with lethal phenotypes upon silencing.

VIGS methodology coupled with biochemical and genetic analyses has been successfully used to functionally characterize plant genes involved in fruit ripening, cell wall composition and plant defense against pathogens and insects [5-12]. Consequently, VIGS has become a powerful reverse genetic tool for functional genomics of crop plants that are not amenable to genetic transformation. Several VIGS vectors have been developed [4,13]. Once developed, VIGS vectors are easy to use and the assays require minimal labor, making the technology ideally suited for high throughput screening of a large number of genes. Currently, soybean transformation procedures are time consuming and cumbersome. A DNA-based VIGS vector was recently developed using *Bean pod mottle virus* (BPMV) [14,15]. This vector was successfully used for the study of soybean genes involved in resistance to Asian soybean rust [16,17]. Recently available soybean genome sequence information [18] and the increasing importance of soybean as a food crop, has led to a tremendous interest in identifying the genes essential for defense against various pathogens, abiotic stress tolerance, and nutritional quality.

Here our aim was to develop an SCN-BPMV-VIGS protocol to extend the use of VIGS for functional analysis of soybean genes involved in resistance to SCN, which is the number one pathogen of soybean in the United States. SCN feeds on the roots of soybean and causes annual yield losses worth more than one billion dollars in the United States alone. To date, VIGS protocols developed for use in soybean have focused on silencing of genes in foliar tissues. Therefore, it is desirable to have a methodology to study loss of function of genes in roots. Here, we describe a VIGS method suitable for gene silencing in soybean roots and demonstrate the methodology by successful silencing of a gene involved in SCN resistance.

## Methods

### Construction of VIGS plasmids

BPMV is a positive strand RNA virus belonging to the Comoviridae family. The virus has a bipartite genome. BPMV vectors were previously made by cloning RNA1 (essential for viral replication and maintenance; pBPMV-IA-R1M) and RNA2 (pBPMV-IA-R2) in plasmid vectors [14] (Note 1). The BPMV-R2 vector used in this study, pBPMV-IA-D35, was modified from the vector described in [14] by engineering *BamH*1 and *Kpn*I cloning sites between the cistrons encoding the viral movement protein and the large coat protein subunit. Inserts are cloned in frame with the viral genes. An extra ‘T’ is introduced in the reverse primer to maintain the reading frame. We used a 328 bp fragment corresponding to the 5’ region of a soybean serine hydroxymethyltransferase (*SHMT*) gene (bps 210-537*; GmSHMT* cDNA sequence, Genbank Accession No. JQ714080) to generate an *SHMT*-VIGS (pBPMV-SHMT) construct. The BPMV vector without an insert (pBPMV vector only) was used as a control.

### VIGS tissue preparation

The VIGS inoculum used in this protocol is generated from infected leaf tissues. Seven-day-old soybean seedlings are used for biolistic delivery of virus vector DNA into leaf tissues. For this, seeds are germinated in Sunshine MVP mix (SunGro Horticulture, Bellevue, WA) in 36-well insert trays at 26°C. Soybean seeds of two recombinant inbred lines, EXF67 and EXF63, derived from a cross between the soybean cultivars Essex and Forrest, were used (Note 2). EXF67 and EXF63 are resistant and susceptible, respectively, to the soybean cyst nematode inbred population PA3 (Hg Type 0). On the day of bombardment, the wells are separated and each well (pot) containing a single healthy seedling with unifoliate leaves is used for biolistic transformation. To generate infected tissues, plasmid DNA encoding RNA1 is co-bombarded into soybean leaves with either RNA2 pBPMV-SHMT or pBPMV vector only using a BioRad® PDS-1000/He biolistic transformation system (BioRad, USA) using the following procedure:

• Prepare DNA by plasmid prep.

• Coat 1 μm gold particles (BioRad cat. No. 165-2263) with 5 μg of DNA (R1:R2; 2 μg:3 μg) as described in the following steps:

 •Place one soybean seedling at a time in the gene gun chamber with unifoliate leaves supported by a plexiglass plate and a wire mesh placed on top to hold the leaves in place. Apply vacuum.

• Bombard the leaves when vacuum reaches 28-inch Hg. Take out the plant, immediately spray with a mist of water. Keep the plants in a tray with a plastic dome cover to maintain humidity. Later, move the tray to 20°C and maintain overnight.

• The plants are then transplanted to 10 cm diameter plastic pots with Sunshine MVP mix and kept at 20°C. Cool temperatures are optimal for virus replication and movement within the plant and symptom development. Water the plants every day and fertilize plants once every week.

• Virus symptoms appear as a mild mosaic of dark and light green regions on leaves and are typically detected on 2^nd^ trifoliate leaves and thereafter. Two to three weeks after bombardment, harvest leaves with virus symptoms, lyophilize and store at -20°C.

1. Sonicate 50 μl gold particles (Note 3) briefly for 15-30 sec. Add 5 μg DNA (volume ≤ to 5 μl) to 50 μl gold particles.

2. Vortex at low speed. While vortexing, add 25 μl 2 M CaCl_2_ and 10 μl 0.1 M spermidine. Vortex for 10 min.

3. Allow to sit for 30 sec.

4. Centrifuge at 1400 rpm in a microfuge and discard the supernatant.

5. Add 140 μl 100% ethanol and re-suspend the gold particles by vigorous tapping. Centrifuge again to remove supernatant.

6. Repeat the ethanol wash two more times.

7. Finally re-suspend the DNA-coated gold particles in 28 μl of 100% ethanol.

8. Vortex and mix the gold particles. While vortexing, pipette 10 μl volume and spread on the middle of three macrocarriers (BioRad cat. No. 165-2335). Allow the ethanol to evaporate.

9. Assemble the gene gun with 1100 psi rupture disk (BioRad cat. No.165-2329), macrocarrier with coated gold particles and stop screen (BioRad cat. No. 165-2336).

**Figure 1:**
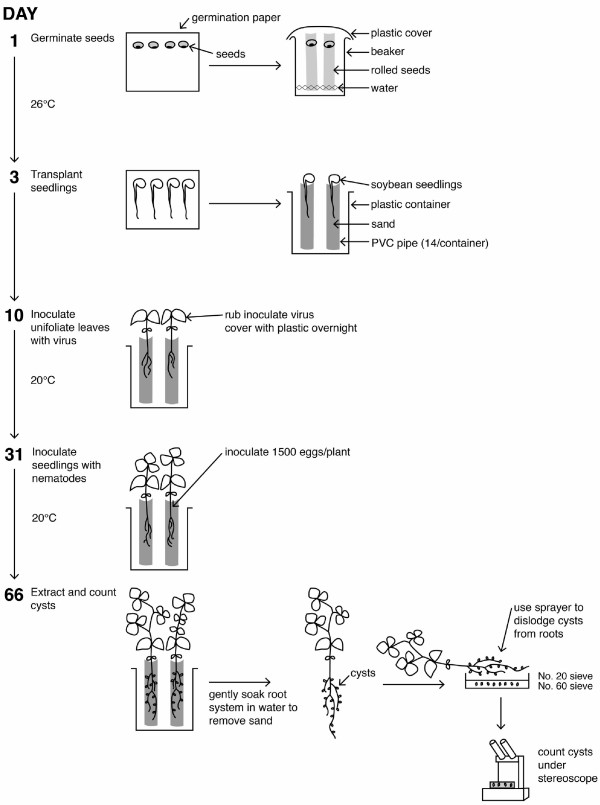
Diagram depicting the virus-induced gene silencing protocol used to test the role of soybean genes involved in interactions with soybean cyst nematode.

**SCN-BPMV-VIGS Protocol** (see Figure 1 for overview)

•(Day 1). Seed germination (2 days). Surface-sterilize seeds with 10% v/v household bleach (0.5% sodium hypochlorite) for 10 min. Wash the seeds with running tap water for 30 min to remove residual bleach. Cut the required number of pieces of germination paper (18” × 7.5”) to roll ragdolls. Fold the germination paper in half and label the fold so the label appears on the outside when rolled. Wet the germination paper with tap water. Open the folded paper (like you would a book) and arrange about 10 seeds on the right side of the paper, in a row 1” from the top edge of the germination paper with hilum facing downward. Fold the left side over as you would close a book. Roll the germination paper from open ends tight enough to hold the seeds in place (approximate diameter will be 1 inch). The label should be seen on the outside of the ragdoll. Place the ragdolls upright in a 1-L beaker with a few milliliters of water. Cover the beaker with saran (plastic) wrap and then poke a few small holes in the wrap with a dissecting needle. Place the beaker of seeds into a 27°C incubator in the dark for 48 h.

•(Day 3). After 48 h, remove the germinated seedlings from 27°C incubator. Select seedlings with a healthy white radicle at least 4-6 cm in length and transplant into a 6 cm hole made in sterile river sand in 100 cm^3^ thin-walled polyvinyl carbonate (PVC) tubes (6” in length, 1-1/8” inside diameter) placed into a 6 qt. Bains Marie plastic container (Continental Carlisle, Oklahoma City, Oklahoma). Fourteen PVC tubes fit into a single container. One plastic container (14 plants) is used per construct to avoid virus contamination among plants infected with different VIGS constructs.

•(Day 3-10). Maintain the plants in a growth chamber set at 20°C with 16 h light, 8 h dark regime and 100-110 μmol cm^-2^ s^-1^ light intensity (7 days) (Note 4).

•(Day 10-31). Virus inoculation (21 days). To prepare inoculum, grind lyophilized BPMV-infected leaf tissue in 50 mM potassium phosphate buffer pH 7.0 (mix 50 mM KH_2_PO_4_ and 50 mM K_2_HPO_4_ in 3.85:6.15 ratio to achieve pH 7.0). Use 25-50 mg dry tissue/ml of the buffer (Note 5). Sprinkle carborandum on the surface of the unifoliate leaves to be inoculated. Gently wipe the leaf surface with cheesecloth 2-3 times in one direction to inflict minute wounds. Next use a fresh piece of cheesecloth wetted in inoculum (2-3 ml is sufficient to infect 14 plants) and gently rub across the leaf surface. Alternatively, you can rub the inoculum on the leaf surface with a gloved finger. Water plants immediately after inoculation and cover with a plastic bag to maintain humidity overnight. This will prevent the wound inoculated leaves from temporary wilting. Maintain the plants for 21 days in a growth chamber set at 20°C with 16 h light, 8 h dark regime and 100-110 μmol cm^-2^ s^-1^ light intensity. Water the plants daily and fertilize weekly with Miracle-Gro (Scotts Miracle-Gro, Marysville, Ohio).

•(Day 31-66). SCN inoculation (35 days; Note 6). Extract cysts of *Heterodera glycines* from infested soil by flotation in water and collect on a no. 60-mesh (250-μm) sieve. Crush harvested cysts gently using a drill press and collect the eggs on a no. 500-mesh (25-μm) sieve [19]. Purify the eggs by centrifugal flotation on a sucrose density gradient and resuspend in water at a concentration of 750 eggs/ml. Using a pencil, poke two 2-2.5 cm deep holes in the soil close to the base of the stem on each side of the plant. Apply 1 ml of inoculum to each hole using a 1 ml pipette (1500 eggs/plant). Maintain the plants for 35 days in a growth chamber set at 20°C with 16 h light, 8 h dark regime and 100-110 μmol cm^-2^ s^-1^ light intensity. Water the plants daily and fertilize weekly with Miracle-Gro (Scotts Miracle-Gro, Marysville, Ohio).

•(Day 66). SCN cyst count. At 35 days post-inoculation, soak each PVC tube in a liter of water until the tube can easily slide away from the root. Gently agitate each root system in the water until the root is free of sand. Using a water sprayer, blast the root system on top of a no. 20-mesh (750 μm) sieve stacked on top of a no. 60-mesh sieve to collect SCN females/cysts. Rinse cysts into 60 mm Petri dishes and manually count under a dissecting microscope. Analyze the treatment and controls for statistical significance using an unpaired two tailed *t*-test. Here, statistical analysis was performed using Graphpad® Prism software. Data were graphed as a vertical scatter plot and analyzed statistically using an unpaired two-tailed *t*-test.

### RNA analysis

Verification of gene silencing is determined by qPCR analysis. The target gene transcript level in a root sample representative of two plants selected from each treatment is analyzed at the time of nematode inoculation (Day 31; 21 days post-virus inoculation).

•Randomly select two plants from each treatment and soak each PVC tube in a liter of water until the tube can easily slide away from the root. Wash each root system in water until the root is free of sand. For RNA extraction and analysis of silencing, harvest tissue from the middle of the root system to the tip. Combine the two root samples for each treatment, flash freeze in liquid N_2_, and store at -80°C.

•Extract total RNA from 100 mg of root tissue using the RNeasy Plant Mini Kit (Qiagen, Valencia, CA) and remove contaminating DNA by on-column DNase I digestion.

•Total RNA can be quantified using a nanodrop ND-1000 (ThermoScientific,Wilmington, DE) and quality assessed by analysis on a 2% agarose gel.

•Synthesize cDNA using the Superscript III cDNA Synthesis Kit (Invitrogen, Grand Island, NY) according to the manufacturer’s instructions.

•Perform qPCR analysis. We used the Applied Biosystems 7500 Real-Time PCR System. Gene-specific primers were designed using the Primer Express software (Applied Biosystems, Carlsbad, CA). Cycling parameters were as follows: 50°C for 2 min, 95°C for 10 min, and 40 cycles of 95°C for 15 s and 60°C for 1 min. A soybean ubiquitin gene [Genbank:D28123] was used as an endogenous control gene for normalization of samples.

Note 1: VIGS vector requests should be made to John Hill (johnhill@iastate.edu) or Steve Whitham (swhitham@iastate.edu), Iowa State University, Ames, IA.

Note 2: Soybean cultivars differ in susceptibility to BPMV infection. Soybean cultivar Williams 82 is highly susceptible. The recombinant inbred lines (EXF63, EXF67) used in our study were less susceptible to BPMV than Williams 82. Thus, the silencing efficiency attained can vary between cultivars.

Note 3: Preparation of gold particles: Weigh 30 mg gold particles (BioRad cat. No. 165-2263), resuspend in 100% ethanol in a 1.5 ml tube, vortex well and let it sit for 10 min at room temperature. Centrifuge the suspension and discard the supernatant. Add 1 ml sterile water, vortex well and centrifuge again to pellet the gold particles. Repeat this washing 2 more times. Add 0.5 ml sterile 50% v/v glycerol to the final pellet of gold particles and vortex well to re-suspend. Finally, while vortexing at low speed, aliquot 50 μl into several 0.5 ml tubes and store at -20°C. Each aliquot is enough to coat DNA that can be used for three separate bombardments.

Note 4: Alternatively, seeds can be germinated, and seedlings grown at 26°C, up until the day of virus inoculation. This will reduce the time to the two-leaf stage by 1-2 days.

Note 5: The amount of tissue required depends on the virus titer of the infected leaf tissues used for inoculum preparation. In our experience, a ratio of 1:20 w/v of tissue results in reliable virus infection on plants. Fresh tissues can also be used, however, more tissue per ml of buffer (1:10 w/v) is recommended.

**Figure 2:**
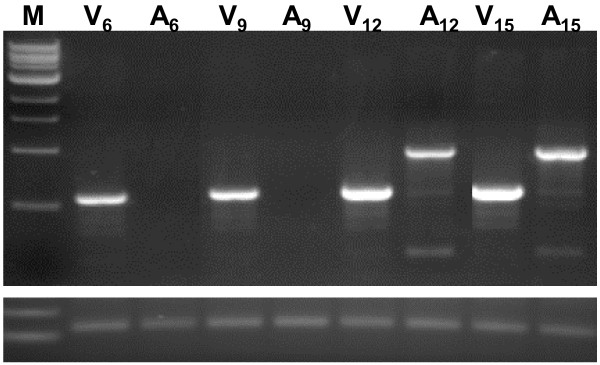
**RT-PCR analysis to detect BPMV in the roots of soybean plants.** RNA was prepared from roots of plants at different days post virus (dpv) inoculation of leaves and used for RT-PCR analysis with BPMV-specific primers. Lanes: M, 1 kb ladder (NEB); V, BPMV only; A, BPMV-*Actin*. Subscripts denote the dpv. Bottom panel, soybean ubiquitin gene [Genbank:D28123] internal control.

**Figure 3:**
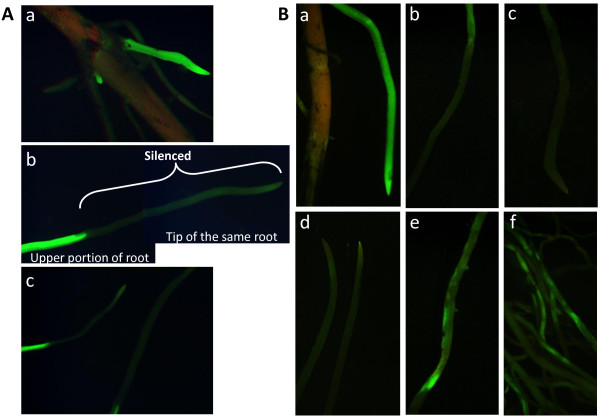
**GFP silencing in transgenic hairy roots expressing GFP using BPMV-GFP-VIGS. GFP silencing was monitored 10 to 25 days post virus (dpv) inoculation. A**. GFP-VIGS at 14 dpv. **(a)** control roots showing GFP expression under control of a soybean ubiquitin promoter [24], **(b)** and **(c)** silenced roots. **B.** GFP-VIGS at 19 dpv.** (a)** control hairy roots, **(b)**-**(d)** root tips showing VIGS silencing, **(e)**-**(f)** more mature roots showing unevenly distributed silencing.

Note 6: We have carried out experiments to determine the time point at which optimum virus levels are achieved in the roots. In a time-course RT-PCR analysis of root tissues harvested at different days post-virus (dpv) inoculation of leaves, we detected BPMV in roots by 6 dpv (Figure 2; V_6_). BPMV carrying a portion of a soybean *Actin* gene (BPMV-*Actin*) was detected in roots at 12 dpv (Figure 2; A_12_). We also examined the timing of *GFP* gene silencing in composite plants [20] expressing GFP in hairy roots. By 14 dpv inoculation we could detect silencing of GFP in roots and the silencing increased by day 19 (Figure 3). Based on these data, we selected 21 dpv inoculation as the optimum timepoint for SCN inoculation. This time scale has also been demonstrated in roots of stable transgenic soybean plants expressing GFP [21].

## Findings

**Figure 4:**
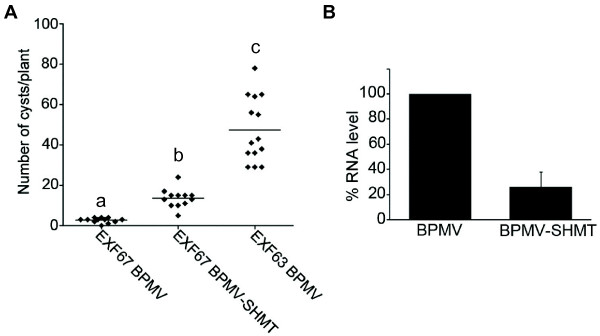
**Soybean cyst nematode development in soybean roots silenced for *SHMT *using virus-induced gene silencing.** EXF67 and EXF63 are SCN-resistant and SCN-susceptible RILS, respectively. Ten days after planting, soybean leaves were rub-inoculated with either BPMV (*Bean pod mottle virus*) or BPMV containing a fragment of the *SHMT* gene sequence (BPMV-*SHMT*). Diamonds represent the number of cysts on a single root system at 35 days post-inoculation. At least twelve plants per genotype treatment were used. Cysts were collected on a 250 μM sieve and counted manually under a dissecting microscope. Six independent experiments were performed showing similar results. Data from one experiment are presented. Different letters denote a significant difference at *P* <0.0001. (B) Effect of BPMV-*SHMT* on *SHMT* transcript levels in soybean roots of ExF67. Reprinted from reference [23] with permission from *Nature*.

Here we present the detailed description of a SCN-BPMV-VIGS pipeline to demonstrate the usefulness of VIGS methodology in studying genes involved in soybean-nematode interactions. A 328 bp fragment corresponding to a *SHMT* gene mapped to the *Rhg4* locus, a major quantitative trait locus for SCN resistance [22], was used for our VIGS experiments. Plants were analyzed at 21 dpv inoculation for RNA silencing. qPCR analysis demonstrated an average of 70% reduction of RNA levels (Figure 4). VIGS plants were infected with SCN at 21 dpv inoculation and the cysts were counted 35 days after SCN inoculation. *SHMT*-VIGS silenced plants showed a statistically significant increase in susceptibility to SCN compared to control plants inoculated with BPMV empty vector in repeated experiments (Figure 4). These experiments demonstrate the successful silencing of a gene involved in SCN resistance. The RNA silencing varied from 65-74% and is as expected for VIGS silencing. The *SHMT* gene has been shown to be required for SCN resistance by TILLING, RNAi, and complementation analysis [23].

## Conclusions

Although VIGS is routinely used for functional studies of genes involved in aboveground interactions, the use of VIGS to study belowground interactions is limited. The development of an efficient VIGS protocol is challenging, in particular when you are dealing with root-biotic interactions. This is due to the fact that inoculation of the virus needs to be carefully timed with inoculation of the infecting organism so that optimal target gene silencing is achieved at the time of infection. In addition, the temperature optimums for the virus and infecting organism must be considered. While our methodology combined established SCN phenotyping and soybean VIGS methods, several specific modifications were made to overcome these challenges. We first analyzed for the presence of BPMV in roots and then monitored silencing of GFP in transgenic hairy roots and the roots of stable transgenics [21] utilizing a GFP-VIGS construct. This allowed us optimize the timing of SCN inoculation of roots (21 dpv) relative BPMV inoculation of foliar tissues. Normally SCN bioassays are performed at 27°C. Since a lower temperature (20°C) is optimal for BPMV, we conducted SCN bioassays at this lower temperature. To compensate for developmental delays of SCN, we extended the SCN bioassay by five days (from 30 to 35 days).

VIGS is a valuable tool for functional genomic studies in crop plants like soybean, where whole plant transformation is difficult and time consuming. This has been validated by recent discoveries of genes involved in Asian soybean rust–soybean interactions [16,17]. Our methodology for VIGS in soybean roots described here can be adapted for genes involved in host responses to other root pathogens, symbiosis, mineral nutrition, abiotic stress responses, root development, among other functions. The enhancements recently made to existing VIGS vectors [15] could potentially improve the silencing further. Additionally, different methods of inoculation can be tested for increased efficiency of silencing and faster screening of candidate genes. One limitation of VIGS is that a negative result cannot rule out a role for a particular gene in a certain function because gene silencing is never 100%. In such cases, VIGS experiments must be combined with other reverse genetic approaches such as RNAi or mutagenesis.

## Competing interests

The authors declare that they have no competing interests.

## Authors’ contributions

PKK, PSJ, TJB, and MGM conceived and designed the study. PKK and RH developed the SCN-BPMV-VIGS pipeline. GY performed the qRT-PCR analysis to detect BPMV in roots, PKK and NWG performed the composite plant assays to monitor GFP silencing in roots, PKK and RH carried out the SHMT-VIGS analysis. JH and SAW provided materials and intellectual input for VIGS analysis. PKK and MGM wrote the manuscript. All authors critically revised and approved the final manuscript.
